# Pharmacokinetics and Target Attainment of SQ109 in Plasma and Human-Like Tuberculosis Lesions in Rabbits

**DOI:** 10.1128/AAC.00024-21

**Published:** 2021-08-17

**Authors:** Oluwaseun Egbelowo, Jansy P. Sarathy, Kamunkhwala Gausi, Matthew D. Zimmerman, Han Wang, Gert-Jan Wijnant, Firat Kaya, Martin Gengenbacher, Nhi Van, Yonatan Degefu, Carol Nacy, Bree B. Aldridge, Claire L. Carter, Paolo Denti, Véronique Dartois

**Affiliations:** a Division of Clinical Pharmacology, Department of Medicine, University of Cape Town, Cape Town, South Africa; b Center for Discovery and Innovation, Hackensack Meridian Health, Nutley, New Jersey, USA; c Department of Molecular Biology and Microbiology, Tufts University School of Medicine, Boston, Massachusetts, USA; d Sequella, Inc., Rockville, Maryland, USA; e Hackensack School of Medicine, Hackensack Meridian Health, Nutley, New Jersey, USA

**Keywords:** *Mycobacterium tuberculosis*, MDR-TB, SQ109, lesion penetration, pharmacokinetics, PK-PD

## Abstract

SQ109 is a novel well-tolerated drug candidate in clinical development for the treatment of drug-resistant tuberculosis (TB). It is the only inhibitor of the MmpL3 mycolic acid transporter in clinical development. No SQ109-resistant mutant has been directly isolated thus far *in vitro*, in mice, or in patients, which is tentatively attributed to its multiple targets. It is considered a potential replacement for poorly tolerated components of multidrug-resistant TB regimens. To prioritize SQ109-containing combinations with the best potential for cure and treatment shortening, one must understand its contribution against different bacterial populations in pulmonary lesions. Here, we have characterized the pharmacokinetics of SQ109 in the rabbit model of active TB and its penetration at the sites of disease—lung tissue, cellular and necrotic lesions, and caseum. A two-compartment model with first-order absorption and elimination described the plasma pharmacokinetics. At the human-equivalent dose, parameter estimates fell within the ranges published for preclinical species. Tissue concentrations were modeled using an “effect” compartment, showing high accumulation in lung and cellular lesion areas with penetration coefficients in excess of 1,000 and lower passive diffusion in caseum after 7 daily doses. These results, together with the hydrophobic nature and high nonspecific caseum binding of SQ109, suggest that multiweek dosing would be required to reach steady state in caseum and poorly vascularized compartments, similar to bedaquiline. Linking lesion pharmacokinetics to SQ109 potency in assays against replicating, nonreplicating, and intracellular M. tuberculosis showed SQ109 concentrations markedly above pharmacokinetic-pharmacodynamic targets in lung and cellular lesions throughout the dosing interval.

## TEXT

In 2019, around 10 million people contracted tuberculosis (TB) worldwide and more than 1.3 million died of the disease (1). TB accounts for more than one in four antimicrobial resistance fatalities per year. Although worldwide incidence and mortality have slightly declined in the past 2 decades, the COVID pandemic is predicted to reverse this trend (2, 3) and the rapid increase in cases of multidrug-resistant and extensively resistant tuberculosis (MDR- and XDR-TB) from approximately 50,000 in 1997 to 500,000 in 2019 poses a serious clinical challenge (4). Treatment of these resistant forms constitutes a dreadful burden for both patients and health care systems, requiring up to 24 months of therapy with three to seven drugs, several of which cause serious side effects (4). For the first time in decades, however, novel three-drug regimens are emerging for MDR-TB (5), and the drug discovery pipeline is growing at a steady pace (https://www.newtbdrugs.org/pipeline/clinical). Clinical trial paradigms and concepts are seemingly evolving, with a clear trend toward the development of regimens rather than single drugs (6). Recently, trials of “universal” drug regimens, aiming to cure drug-resistant and drug-susceptible TB, have seen promising clinical outcomes (7, 8). Thus, the new challenge faced by clinical developers is to rationalize and optimize the selection of promising safe and novel regimens for testing in TB patients.

SQ109 is a well-tolerated drug candidate that has completed phase II clinical development (9–11). In Mycobacterium tuberculosis, it targets the mycolic acid transporter MmpL3, resulting in inhibition of cell wall biosynthesis (12, 13). Difficulties in isolating resistant mutants and the observation that SQ109 is active against microbial species that lack MmpL3 and mycolic acids (9, 14) pointed toward additional mechanism(s) of action. Accordingly, SQ109 was found to also interfere with menaquinone biosynthesis and electron transport, inhibiting respiration and collapsing the pH gradient and membrane potential (15, 16). SQ109 exhibits MICs ranging from 0.12 to 0.78 mg/liter (17) and is bactericidal within this MIC range (9). It kills 90% of nonreplicating M. tuberculosis under low oxygen conditions at 1.1 mg/liter and is also bactericidal against the streptomycin-dependent M. tuberculosis 18b strain, a tool for assessing drug potency against nonreplicating bacteria, albeit at a significantly higher concentration of 10 μg/ml (18). At its MIC, it reduces growth of intracellular M. tuberculosis by 99% (17). Consistent with its membrane-targeting mechanism of action, it potentiates bedaquiline, an inhibitor of ATP synthase (16). It also synergizes with rifampicin both *in vitro* (19) and in TB mouse models (20, 21). The observed synergy of SQ109 with rifampicin in mice led to the design of two clinical trials in which SQ109 was combined with rifampicin—an early bactericidal activity (EBA, ClinicalTrials registration no. NCT01218217) trial (22) and a phase II 3-month trial (MAMS, ClinicalTrials registration no. NCT01785186) (10). While a drug-drug interaction between SQ109 and rifampicin identified in the EBA study and confirmed in the MAMS trial (22, 23) likely contributed to disappointing treatment-shortening results, the percentage sputum clearance of all MAMS arms at week 12 ranged from 96 to 98% with no difference between arms. Interestingly, a reanalysis of sputum from the EBA study, using metabolic dyes to sort M. tuberculosis into viable replicating, viable not replicating, and nonviable bacteria (24), demonstrated that the addition of SQ109 to rifampicin significantly increased the reduction in viable CFU and prevented an increase in Nile red-positive persister cells, described as viable but not replicating bacteria (24). This observation is consistent with findings in mice from a variety of laboratories (18). Recently, SQ109 was evaluated in a phase IIb/III MDR-TB clinical trial in Russia, designed to determine if SQ109 added to the MDR-TB standard of care (SOC, 5 drugs specified by the Russia Ministry of Health) could improve sputum clearance by 25% in 6 months compared to SOC plus placebo. SQ109 met both safety and efficacy endpoints, as summarized in Table 1 since the trial results were published in Russian (11). SQ109 activity against MDR-TB was similar to that observed in registration trials of bedaquiline (25) and delamanid (26).

**TABLE 1:** Summary of SQ109 phase IIb trial in MDR-TB patients in Russia^*a*^ conducted under ICH guidelines (11) and comparison of clinical outcomes in similar trials with bedaquiline and delamanid

Drug	8-wk sputum clearance (%)		24-wk sputum clearance (%)			Median time	
	Drug-treated	Placebo	Drug-treated	Placebo	*P* value (risk ratio)	Drug-treated	Placebo
Bedaquiline (25, 70)	48	9	78	58	<0.014 (2.44)	8 wks	18 wks
Delamanid (26, 71, 72)	42	30	75	55	<0.001 (1.35)	Not reported	Not reported
SQ109 (11)	52	38	80	61	<0.041 (2.47)	8 wks	12 wks

a English abstract from reference 11: “A multi-center, double, blind, randomized, placebo-controlled study was conducted in two parallel groups from September 21, 2012 to September 30, 2016, in 6 research centers located in 5 cities of the Russian Federation. Main results. 1. Cessation of bacillary excretion confirmed by cultures on liquid media by the end of the 6th month of the intensive phase of chemotherapy in pulmonary MDR TB patients receiving SQ109 was observed confidently more often versus treatment regimens containing only existing anti-tuberculosis drugs: both for ITT population (61.0% versus 42.9%, *P *= 0.0412), and PP population (79.7% versus 61.4%, *P *= 0.0486). 2. There were no statistically significant differences in the achievement of sputum conversion between the groups, but by the end of the 8th week, the sputum converted in 52% of patients in the group treated with SQ109 versus 38% in the group taking a placebo. The median time of bacillary excretion cessation confirmed by culture on liquid media in SQ109 group made 56 days, while in the placebo group it was 84 days. 3. Use of SQ109 along with basic chemotherapy for pulmonary MDR TB did not result in the higher frequency of adverse events, worsening of their severity, development of new variants of adverse events compared to the basic anti-tuberculosis treatment of this group of patients in combination with placebo. 4. Results of the study allow concluding that SQ109 is an effective drug, satisfactory tolerated (compatible with tolerability of placebo) being a part of integral etiotropic chemotherapy of pulmonary MDR TB patients.”

Drug distribution and efficacy studies in animal models of TB disease show that reaching adequate drug concentrations at the site of infection is critical to achieve sterilization (27–30). Inadequate coverage of specific areas at the site of disease may contribute to emergence of resistance, particularly in lesion compartments that are poorly or not vascularized, such as cavity caseum and the necrotic core of granulomas (27, 29–32). Therefore, measurement, modeling, and simulation of drug distribution in lesions is critical to prioritize new regimens that achieve optimal lesion coverage.

Despite relatively low oral bioavailability (16) and first-pass liver metabolism (33), SQ109 distributes favorably in all organs, with significant accumulation in mouse lung and spleen tissue well above its MIC (34). This leads to a long elimination half-life, in excess of 60 h, in humans (9). However, distribution in TB-infected lung and mature necrotic lesions has not been assessed to date.

The present study dissects the sterilization potential of SQ109 in TB lesions, with the objective of guiding its inclusion in drug regimens that leverage its activity. We used the rabbit model of active TB to quantify penetration of SQ109 in cellular and caseous lesion areas and relate these values to potency metrics determined *in vitro* against extracellular, intracellular, and nonreplicating M. tuberculosis bacilli, alone and in combination with other TB drugs. Pharmacokinetic-pharmacodynamic (PK-PD) modeling was used to visualize target attainment in uninvolved lung, in the granuloma cellular rim, and in caseous foci of lung lesions. These results will be critical to design SQ109-containing regimens that maximize the drug’s contribution to lesion sterilization.

## RESULTS

### *In vitro* partitioning of SQ109 in TB lesions.

**FIG 1:**
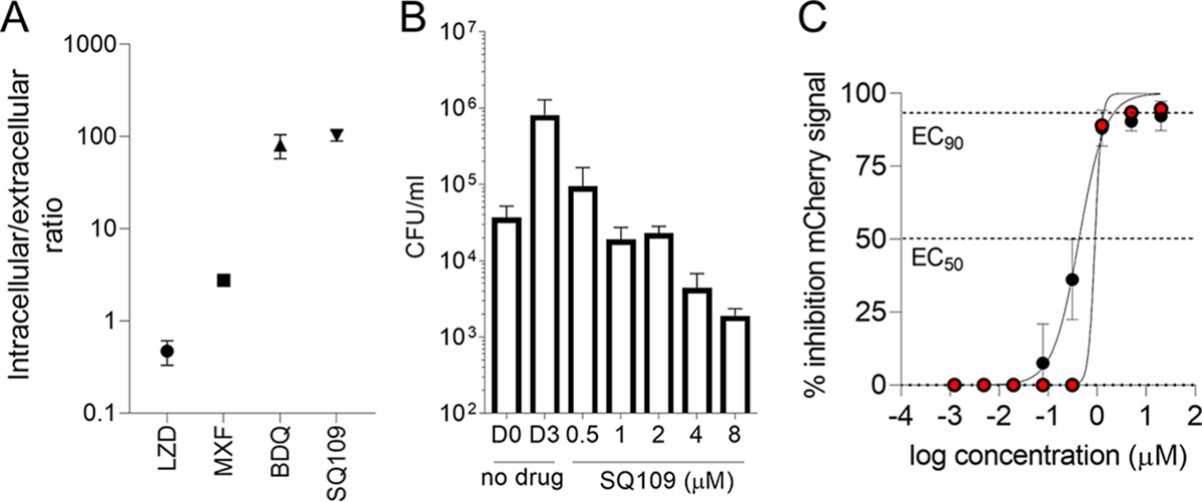
*In vitro* profiling of SQ109 uptake and activity in macrophages. (A) Uptake into THP-1 derived macrophages compared to control TB drugs representative of low (linezolid, LZD), medium (moxifloxacin, MXF), and high (bedaquiline, BDQ) uptake. Mean intracellular to extracellular ratio after 30 min of incubation and standard deviations are shown (*n* = 3). (B) Bactericidal activity of SQ109 against M. tuberculosis in THP-1-derived macrophages exposed to indicated concentrations for 3 days. D0 and D3, intracellular bacterial burden expressed as CFU per ml prior to SQ109 addition and on day 3 in the drug-free control well. The experiment was carried out two times independently, with technical triplicates, and one representative data set is shown. (C) Dose-response effect curve of SQ109 against intracellular M. tuberculosis in human monocyte-derived macrophages under normoxic (black circles) and hypoxic (red circles) conditions. Differentiated macrophages were infected with M. tuberculosis Erdman carrying an mCherry red fluorescent protein reporter for 4 h, washed, and incubated under normoxic or hypoxic conditions for 3 days prior to addition of SQ109 for 5 days. The experiment was carried out three times independently, with technical triplicates, and one representative data set is shown.

We observe that different drug classes penetrate differentially into the two major compartments of TB granulomas where the pathogen resides—the cellular cuff made of phagocytes and other immune cells and the nonvascularized necrotic core (29). To predict the partitioning of SQ109 between these two critical sites of infection, we applied previously validated *in vitro* assays of caseum binding and uptake into macrophages (29, 35). The average caseum-free fraction (caseum *f*_u_) of 0.03 to 0.06% (standard deviation [SD], 0.01 to 0.02%) was much lower than protein binding in plasma (*f*_u_ range of 77 to 94%) measured across species (33). In THP-1-derived macrophages, the average intracellular to extracellular (I/E) concentration ratio after 30 min of drug exposure was approximately 100. Comparative I/E ratios of second-line TB drugs that fall in the low, medium, and high macrophage uptake category are provided as a reference (Fig. 1A). SQ109’s high caseum binding and high uptake into macrophages was comparable to those of bedaquiline and was predictive of steep concentration gradients decreasing from the cellular rim into the necrotic core of granulomas (36).

This high macrophage uptake and low plasma level of SQ109 in humans and preclinical species (22, 33) prompted us to measure SQ109’s partitioning in human blood cells. Interestingly, we found close to 3:1 red blood cell (RBC) to plasma concentration ratios, and up to 5,000-fold accumulation in mononuclear white blood cells (WBC) compared to plasma. Whole blood concentrations were only 6-fold higher than plasma concentrations despite the large partitioning coefficient in WBC due to the small relative volume of WBC (incubation concentration, 500 nM; whole blood, 314 nM; plasma, 53 nM; intracellular concentration in RBC [reached after 1 h of incubation], 179 nM; intracellular concentration in WBC [reached after 1 h of incubation], 263,866 nM; partition coefficient from plasma to red blood cells [*k*_RBC,p_], 3.4; partition coefficient from plasma to white blood cells [*k*_WBC,p_], 5,016). Overall, we measured consistent high partitioning of SQ109 in THP-1-derived macrophages and in human WBC, suggesting the potential for high concentrations in cellular areas of TB lesions relative to plasma.

### Activity of SQ109 against M. tuberculosis populations relevant to TB lesions.

To place the lesion concentration of SQ109 into a pharmacodynamic context, we measured concentrations required for growth inhibition and killing of intracellular M. tuberculosis in THP-1-derived macrophages and in primary human macrophages derived from blood monocytes. In infected THP-1-derived macrophages treated for 3 days with SQ109, 90% growth inhibition (intracellular IC_90_) was achieved at 0.5 μM or 0.17 mg/liter, and a 1-log kill (intracellular MBC_90_) was achieved at 4 μM or 1.3 mg/liter (Fig. 1B). Compared to the corresponding values for a panel of TB drugs (37), SQ109 showed potent inhibitory and bactericidal activity against intracellular M. tuberculosis. Next, we measured the inhibitory and bactericidal activity of SQ109 against intracellular M. tuberculosis in monocyte-derived macrophages under normoxic and hypoxic conditions, the latter inducing the formation of lipid droplets that leads to slowed bacterial replication, loss of acid fastness, and tolerance to isoniazid and rifampicin (38, 39). Using this model, we infected macrophages with a red fluorescent (mCherry) reporter M. tuberculosis Erdman strain (40, 41), preincubated them under normoxic or hypoxic conditions for 3 days, and then treated them for 5 days with SQ109 at concentrations ranging from 1 nM to 20 μM. Fluorescence was monitored to assess the numbers of remaining bacteria. We found that SQ109 is equally potent under both conditions and inhibits 90% of the red fluorescence signal around 1 μM in hypoxic and normoxic macrophages (Fig. 1C). Thus, SQ109 is as active against slow-replicating M. tuberculosis in hypoxic foamy macrophages—found at the inner cellular border near the necrotic core of granulomas (39)—as it is against replicating M. tuberculosis in normoxic macrophages. Potency values measured in this study and key potency values published elsewhere, which enable PK-PD parameter calculation, are summarized in Table 2.

**TABLE 2:** Potency values against M. tuberculosis populations typically residing in lung lesions^*a*^

Description	PD parameter abbreviation	Value (mg/liter)	Reference(s) or source
Minimum bactericidal concn	rMBC_90_	0.64	9
Minimum inhibitory concn	MIC	0.25–0.78	19, 73, 74
Nonreplicating M. tuberculosis in streptomycin-dependent M. tuberculosis strain	nrMBC_90_	10	18
Nonreplicating M. tuberculosis in low-oxygen recovery assay (LORA)	Low O_2_ MIC or nrMIC_90_	1.1	74, 75
Macrophages THP-1 (growth inhibition)	MacEC_90_	0.4	This work
Macrophages THP-1 (killing)	MacMBC_90_	1.3	This work

a nrMBC_90_, concentration that kills 90% of nonreplicating M. tuberculosis bacilli; nrMIC_90_, concentration that inhibits 90% growth in the recovery phase of the LORA (low-oxygen recovery assay); MacEC_90_, concentration that inhibits 90% of growth in macrophages; MacMBC_90_, concentration that kills 90% of M. tuberculosis bacilli in macrophages; rMBC_90_, concentration required to achieve 90% killing in replicating culture.

Growth inhibitory and bactericidal activity is reported for SQ109 alone and in combination with a small number of selected drugs (19–21, 42). To assess its potential for synergy with drugs included in regimens that have performed well in recent MDR-TB trials, we tested its activity in pairwise combinations with 1st- and 2nd-line drugs using diagonal measurement of *n*-way drug interactions (43) under several conditions that mimic lesion environments—cholesterol or fatty acids as carbon sources, butyrate combined with high nitrate, and reduced oxygen tension to emulate pseudodormancy and induce nonreplication (44). We found previously observed synergistic combinations with drugs such as rifampicin (19) (and, to a lesser extent, rifapentine) and clofazimine (45). These extended to all conditions tested (Table 3). We also found condition-dependent synergy with pretomanid, moxifloxacin, and bedaquiline when valerate was the carbon source. Interestingly, SQ109 synergized with all drugs tested under the 3-stress condition combining fatty acid, nitrate, and low oxygen. These results suggest positive drug combination potential for SQ109 against nonreplicating M. tuberculosis persisters in lung lesions.

**TABLE 3:** Pairwise drug combinations with SQ109 measured using diagonal measurement of *n*-way drug interactions (DiaMOND)

Drug added to SQ109	FIC parameter^*a*^ and growth condition^*b*^^,^^*e*^					
	FIC_75_ standard medium^*c*^	FIC_90_ cholesterol high	FIC_75_ cholesterol^*c*^	FIC_90_ butyrate	FIC_90_ valerate	FIC_90_ butyrate + nitrate (dormancy)
Bedaquiline	0.99	1.74	1.36	1.53	0.77	0.51
Clofazimine	0.73	0.77	0.57	0.62	nr	0.32
Ethambutol	0.94	1.28	1.49	1.50	1.27	NR
Isoniazid	1.44	1.24	1.74	1.01	1.00	0.59
Linezolid	1.15	1.00	0.93	0.93	0.74	0.68
Moxifloxacin	0.74	1.06	1.01	0.79	0.69	0.69
Pretomanid	1.09	1.36	1.49	1.61	0.62	0.69
Pyrazinamide	NR	0.79	0.86^*d*^	1.09^*d*^	0.73^*d*^	NR
Rifapentine	1.04	0.75	0.84	0.63	0.63	0.60
Rifampicin	0.59	0.77		0.73	0.65	0.48

a FIC_x_ is the fractional inhibitory concentration evaluated at IC_x_. FIC of <1 and >1 are indicative of synergy and antagonism, respectively.

b See Materials and Methods for complete description of growth conditions.

c FIC_75_ is provided rather than FIC_90_ under these conditions since several antibiotics did not reach IC_90_ (see Materials and Methods for details).

d IC_75_ was not reached; hence, FIC_50_ values were calculated using IC_50_ values for pyrazinamide.

e NR, not reached.

### Pharmacokinetics and distribution of SQ109 in infected lung and lesions.

To determine the human-equivalent dose and PK parameters of SQ109 in rabbits, we dosed groups of three animals by intravenous and oral routes (see Fig. S1 in the supplemental material). SQ109 had a high volume of distribution, medium to high clearance, and low oral bioavailability, determined using plasma concentrations (Table 4). This had been observed in other animal species and in clinical trials (22, 33). The high clearance (approximately equivalent to liver blood flow) and low bioavailability have been attributed to hepatic metabolism and first-pass clearance in other species. The rabbit dose predicted to best match average *C*_max_ (peak plasma concentration) and AUC (area under the concentration-time curve) in patients receiving 300 mg daily was approximately 25 mg/kg body weight.

**TABLE 4:** Pharmacokinetic parameter estimates of the structural and final model in rabbits

Parameter^*a*^	Estimate	SIR 95% CI^*b*^
CL	7.77 liters/h	5.04–10.9
V_c_	6.04 liters	3.69–8.82
*K_a_*	0.187 liters/h	0.106–0.284
F V_p_ Q Cellular lesion equilibration t_1/2_ Caseous lesion equilibration t_1/2_ Lung equilibration t_1/2_ Caseum equilibration t_1/2_ R_CLH_ R_CAH_ R_LUH_ R_CAL_	2.60% 81.8 liters 28.1 liters/h 16.9 h 8.25 h 4.65 h 25.7 h 4,270 3,130 3,456 138	1.79–3.76 49.8–122 15.8–1.2 11.7–25.7 5.68–11.9 3.09–7.07 15.8–46.2 3,189–5,522 2,370–4,108 2,664–4,523 92.6–204.1
BSV CL (%)	19.7	13–25
BOV F (%)	19.8	13–25
BOV K_a_ (%)	92	65–114
Additive error plasma Proportional error plasma Proportional error cellular Proportional error caseous Proportional error lung Additive error caseum Proportional error caseum Scaling factor cellular lesion^*c*^ Scaling factor lung tissue^*d*^	0.0029 mg/liter 40% 40% 39% 39% 0.0043 mg/liter 49% 9.5% 40%	0.0011–0.0045 28–57 30–56 31–54 30–53 0.0020–0.0067 33–75 07–12 31–49

a CL, drug clearance; V*_c_*, central compartment volume; V*_p_*, peripheral compartment volume; *K_a_*, first-order absorption rate constant; F, oral bioavailability; Q, intercompartment rate of the plasma. K_CLH_, K_CAH_, K_LUH_, and K_CAL_, are the time rate constants for cellular lesion, caseous lesion, uninvolved lung tissue and caseum which are reported as half-life in this table. R_CLH_, R_CAH_, R_LUH,_ and R_CAL_ are compartmental pseudopartition coefficients for cellular lesion, caseous lesion, uninvolved lung tissue, and caseum, respectively. Typical values of clearance and volume of distribution were allometrically scaled with body weight, and the typical values reported are for a rabbit with a body weight of 3.5 kg (mean of all study animals). BSV, between subject variability; BOV, between occasion variability.

b The 95% confidence interval (CI) of parameter estimates computed with sampling importance resampling (SIR) on the final model.

c Scaling factor to convert the measurements from LCM cellular samples to cellular lesion homogenates.

d Scaling factor to convert the measurements from LCM lung samples to uninvolved lung tissue homogenates.

**FIG 2:**
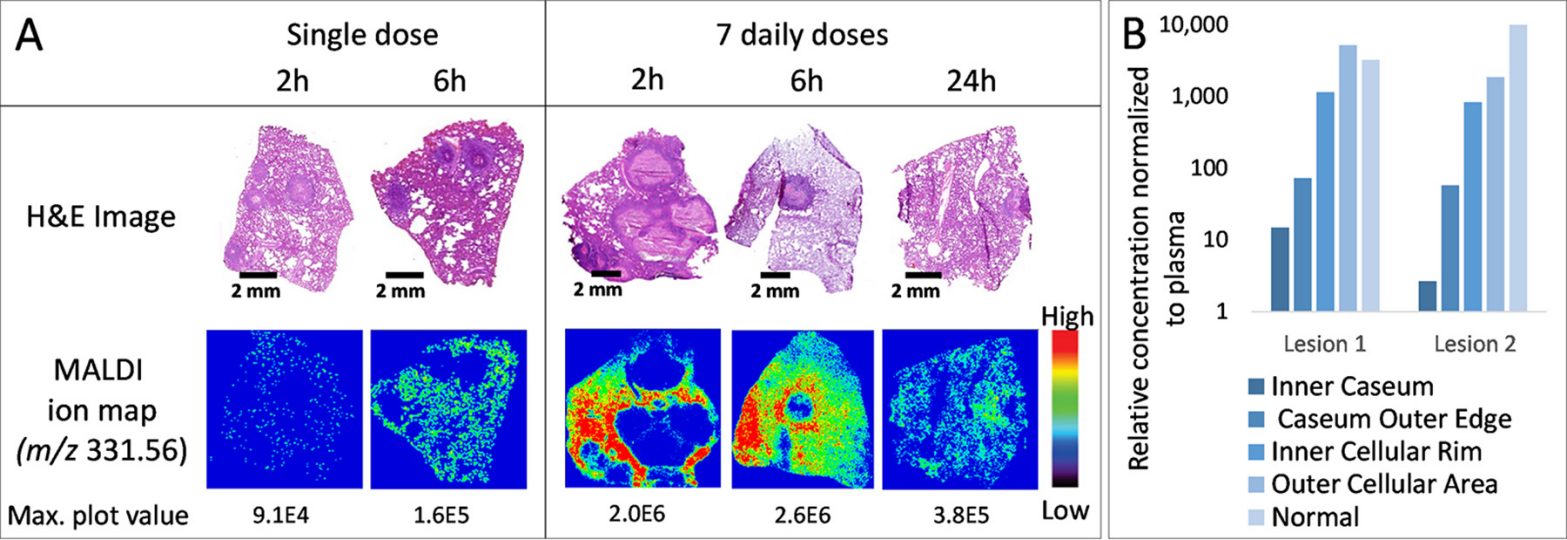
Spatial distribution of SQ109 in necrotic lesions. (A) MALDI MS images of SQ109 [M+H]^+^ in rabbit necrotic lesions and corresponding hematoxylin and eosin (H&E) stained sections. Rabbits received a single dose or 7 daily doses of SQ109 prior to imaging, and lesions were collected 2, 6, and 24 h postdose. The intensity scale is shown to the right of the MS images, and the maximum plot intensity is indicated under each image. (B) Concentration ratios in lesion areas spanning from the core of the caseous region to the uninvolved lung tissue surrounding the lesion, relative to plasma, measured in thin-section samples collected by laser-capture microdissection from two large necrotic lesions as illustrated in Fig. S2. Concentrations were adjusted to account for SQ109 instability and/or limited recovery as described in Materials and Methods. Both lesions were collected from TB-infected rabbits 6 h after the last of 7 daily 25-mg/kg doses.

To image and quantify the distribution of SQ109 in pulmonary lesions *in vivo*, New Zealand white rabbits were infected with M. tuberculosis HN878 for 12 to 16 weeks until they developed mature and diverse lesions. The infected rabbits then received either a single oral dose or seven daily doses of SQ109 at 25 mg/kg either 2 h, 6 h, or 24 h prior to lung and lesion dissection. Representative lesions were sectioned and analyzed by matrix-assisted laser desorption ionization–time of flight (MALDI) mass spectrometry imaging and then stained with hematoxylin and eosin to reveal the underlying cellular composition of the lesion. As predicted by high caseum binding and high uptake in macrophages and WBC, we observed strong signals in the cellular rim of lesions, particularly after 7 doses, and poor or slow diffusion into avascular caseum (Fig. 2A).

All other lesions and lung tissue samples were either (i) homogenized and SQ109 was quantified by high-pressure liquid chromatography coupled to tandem mass spectrometry (LC-MS/MS) or (ii) sectioned for spatial quantitation by laser-capture microdissection (LCM) and LC-MS/MS (46). In total, SQ109 concentrations were measured in 132 plasma samples and 402 lesion or lung tissue samples. Consistent with *in vitro* uptake in macrophages and MALDI ion maps, we found SQ109 concentrations to be approximately 3 orders of magnitude higher in apparently uninvolved lung tissue, which contains microcellular lesions, and in lesion homogenates than in plasma. To quantify the partitioning of SQ109 between cellular and necrotic lesion areas, we sampled these regions in thin tissue sections by LCM and measured SQ109 concentrations by LC-MS/MS. These data confirmed the very high extent of SQ109 penetration in lung and cellular lesion homogenates and the steep decreasing gradient going inward from the cellular rim of granulomas into the caseous core. Results for two representative large necrotic lesions are shown in Fig. 2B and Fig. S2, confirming the trend observed in MALDI ion maps. Overall, total SQ109 concentrations are lowest in plasma (markedly lower than the MIC throughout the dosing interval) and approximately 1,000 to 5,000 times higher in lung tissue, cellular lesions, and the cellular area of necrotic lesions than in plasma. Although much lower in caseum than in areas made of host cells, SQ109 accumulated up to 100-fold in caseum relative to plasma after seven daily doses. These results indicated that both lesion and plasma PK-PD parameters, total and unbound when measurable, should be determined to understand the drivers of SQ109 efficacy.

**FIG 3:**
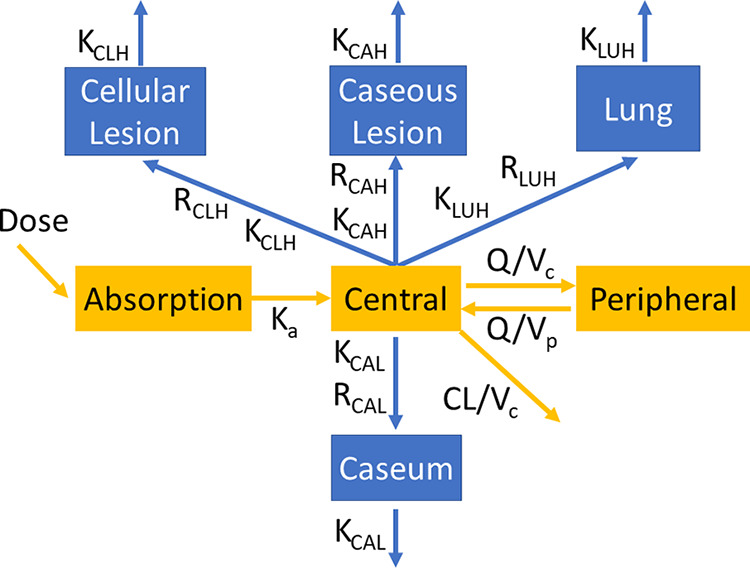
Schematic diagram of the final model. *K_a_*, absorption rate constant; all other *K* values are rate constants for the transfer of drug from plasma to the respective tissue or lesion; CLH, cellular lesion homogenates; CAH, caseous lesion homogenates; LUH, lung homogenates; CAL, caseum LCM samples; Q, intercompartment rate; R, pseudopartition coefficient into the respective tissue compartment; V_c_, volume of distribution in the central compartment; V_p_, volume of distribution in the peripheral compartment; CL, clearance. Scaling factors required to adjust for SQ109 loss during laser-capture microdissection were estimated by the model using the cellular lesion and lung data sets as anchors and applied to the caseum data set as described in the Materials and Methods.

**FIG 4:**
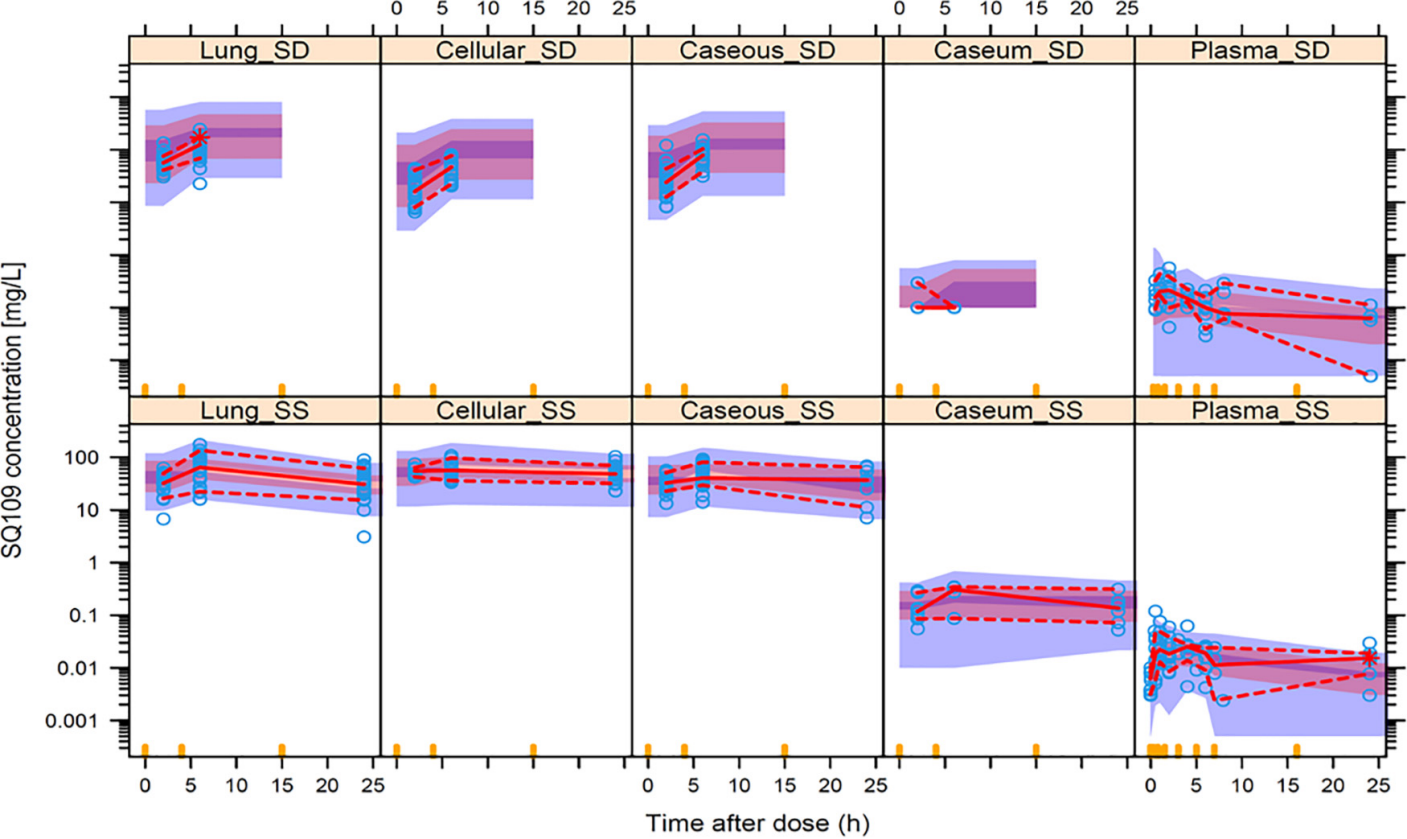
Visual predictive check (VPC) for the final SQ109 population pharmacokinetic model with log-normalized *y* axis scale. Plasma, cellular lesion, caseous lesions, caseum, and lung tissue data sets—generated from homogenates and LCM samples after scaling adjustment—were included. Separate plots of preadjustment data are shown in Fig. S3. Concentration-time profiles following a single dose (SD) and at steady state (SS) are shown. Open circles are observed concentrations. The middle continuous line is the 50th percentile of observed data, and the upper and lower dashed lines are the 95th and 5th percentiles, respectively. The shaded regions represent the 95% prediction intervals of the 10th, 50th, and 90th percentiles.

**Structural pharmacokinetic and lesion penetration model.** Formal modeling of the data set was carried out to calculate PK-PD parameters in key infected compartments, i.e., cellular and necrotic lesions, caseous foci, and apparently uninvolved lung. A depiction of the structural model is provided in Fig. 3. The pharmacokinetics of SQ109 in plasma was well described using a two-compartment disposition model with first-order absorption and elimination. The uninvolved lung tissue and lesion data were modeled with separate “effect compartments” as previously described (27, 47). The final parameter estimates are provided in Table 4. For a typical 3.5-kg rabbit, the estimated clearance (CL) was 7.77 liters/h. Oral absorption was slow and oral bioavailability was low (2.6%), as observed in other species (33). Model estimates of penetration coefficients were 4,270, 3,130, 3,456, and 138 for cellular lesions, caseous lesions, uninvolved lung tissue, and caseum, respectively. SQ109 appeared to equilibrate most rapidly in uninvolved lung tissue (equilibration half-life of 5 h) compared to whole lesions (8 to 17 h) and caseum (26 h). To account for differences in SQ109 recovery in LCM samples compared to tissue homogenates, the structural model was used to estimate scaling factors for uninvolved lung tissue (2.5) and cellular lesions (10). Through Monte Carlo simulations, we next used the model to predict concentration distributions in 1,000 simulated subjects (rabbits) in plasma, uninvolved lung, cellular lesions, caseous lesions, and caseum following a single dose or 7 daily doses of 25 mg/kg. The performance of the final model was assessed by internal validation with visual prediction check (VPC) using data prior to pooling tissue homogenate and LCM sample data (Fig. S3) and after pooling tissue homogenate and LCM sample data postadjustment with scaling factors (Fig. 4). The VPCs indicate an adequate model fit of the data, with data falling within the 95% prediction intervals of the 10th, 50th, and 90th percentiles. The most striking properties of the concentration-time profiles at steady state were the narrow peak-to-trough ratio and the markedly increased accumulation in all tissue compartments after 7 doses compared to a single dose (Fig. 4).

**FIG 5:**
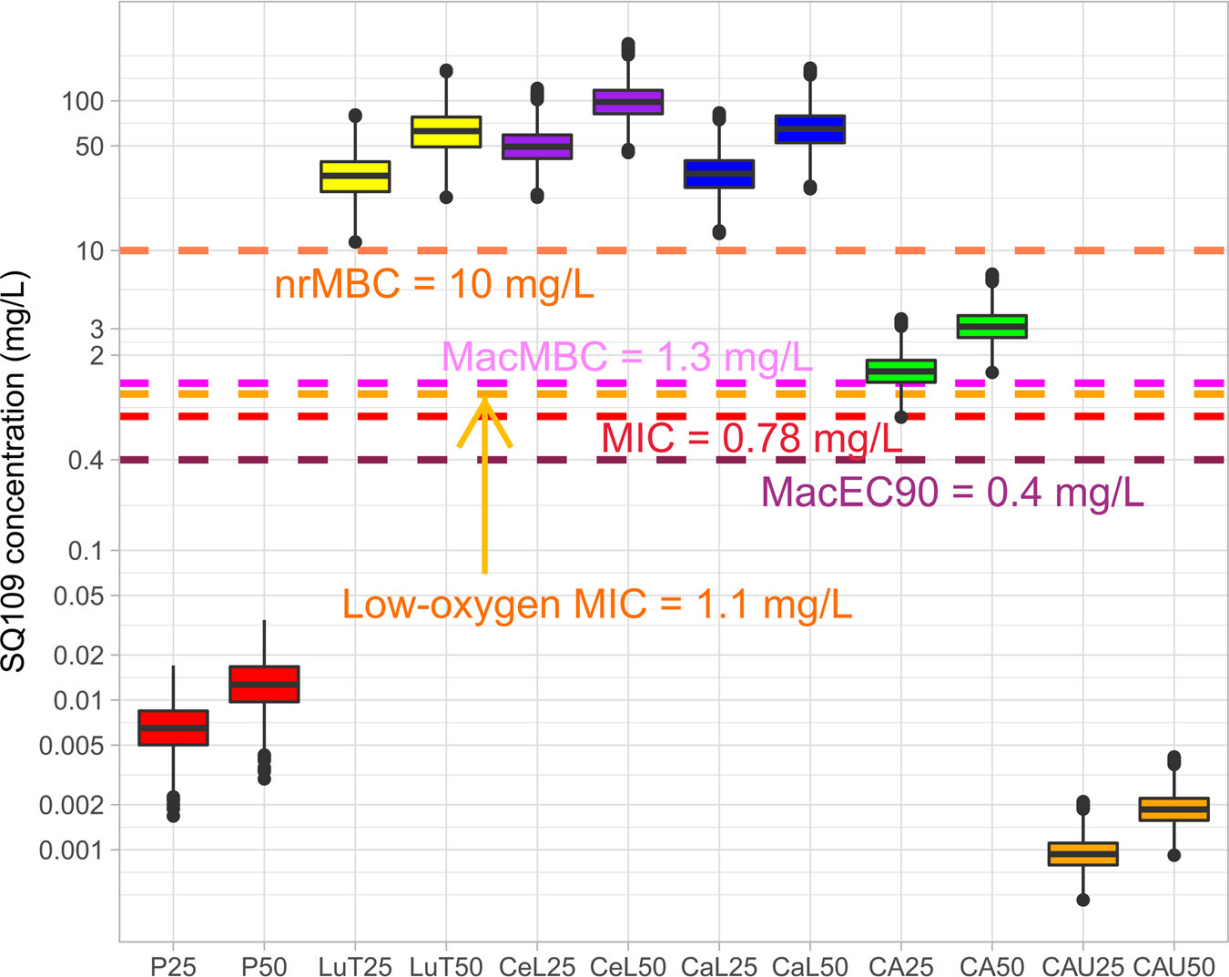
Trough concentration distributions in plasma and lung/lesion compartments in relation to potency values. Simulations of standard SQ109 dose (25 mg/kg in rabbits equivalent to 300 mg daily in patients) and high dose (50 mg/kg in rabbits equivalent 600 mg) were carried out in 1,000 rabbits, and trough distributions are shown as boxes (25th to 75th percentiles) and whiskers (2.5th to 97.5th percentiles) of the simulated concentrations on day 7. Total concentrations are shown unless stated otherwise. P, plasma; CeL, cellular lesion; CaL, caseous lesions; LuT, uninvolved lung tissue; CA, caseum, CAU, caseum unbound. Description of the PD parameters is provided in the text.

**Simulations of SQ109 PK-PD coverage in plasma, lung, and lesion compartments.** To predict lesion PK-PD coverage, we used the structural model to simulate concentration distributions in 1,000 rabbits in plasma, uninvolved lung, cellular lesions, caseous lesions, and caseum following (i) 7 daily doses of 25 mg/kg, reproducing exposure in TB patients receiving the standard 300 mg daily dose, and (ii) 50 mg/kg reproducing simulated exposure in patients receiving 600 mg daily. Given the narrow peak-to-trough fluctuations, we plotted the 95% confidence intervals (95% CI) of trough concentrations in each compartment against the most relevant potency value for the corresponding bacterial populations (Fig. 5). Plasma troughs were compared to standard MIC. Troughs in lung, cellular rims, and cellular and caseous lesions were compared to the MBC_90_ against replicating and nonreplicating bacteria under normoxic and low-oxygen conditions and to inhibitory and bactericidal concentrations against intracellular bacteria in normoxic and hypoxic foamy macrophages (MacEC_90_ and MacMBC_90_). Troughs in caseum were compared to MBC_90_ against replicating and nonreplicating bacteria under normoxic and low-oxygen conditions. All pharmacodynamic parameters measured and/or used for PK-PD assessment are summarized in Table 2. SQ109 troughs did not reach the MIC in plasma, even at the high simulated dose and using total concentrations (the fraction unbound in plasma ranges between 10 and 20% across species [33]). In uninvolved lung and lesions, SQ109 was above all growth inhibitory and bactericidal concentrations against extracellular and intracellular, replicating and nonreplicating bacteria throughout the dosing interval. While assays performed against extracellular M. tuberculosis in standard growth media only partially account for protein binding (7H9 growth medium contains 5% bovine serum albumin fraction V), potency assays against intracellular M. tuberculosis in macrophages contain 10% fetal bovine or human serum, and macrophages largely recapitulate the binding conditions found at the site of infection. In caseum, where nonreplicating M. tuberculosis bacilli reside under low-oxygen conditions, total SQ109 trough concentrations were above the low-oxygen MIC (concentration required to inhibit growth in the low-oxygen recovery assay [LORA]) but did not reach the concentration that kills 90% of nonreplicating M. tuberculosis bacilli (nrMBC_90_). However, SQ109 concentrations corrected for caseum binding (caseum *f*_u_) did not reach the potency targets (Fig. 5). SQ109 activity against M. tuberculosis persisters in *ex vivo* caseum (48) could not be determined due to its instability in this matrix (less than 1% remaining after 7 days).

**FIG 6:**
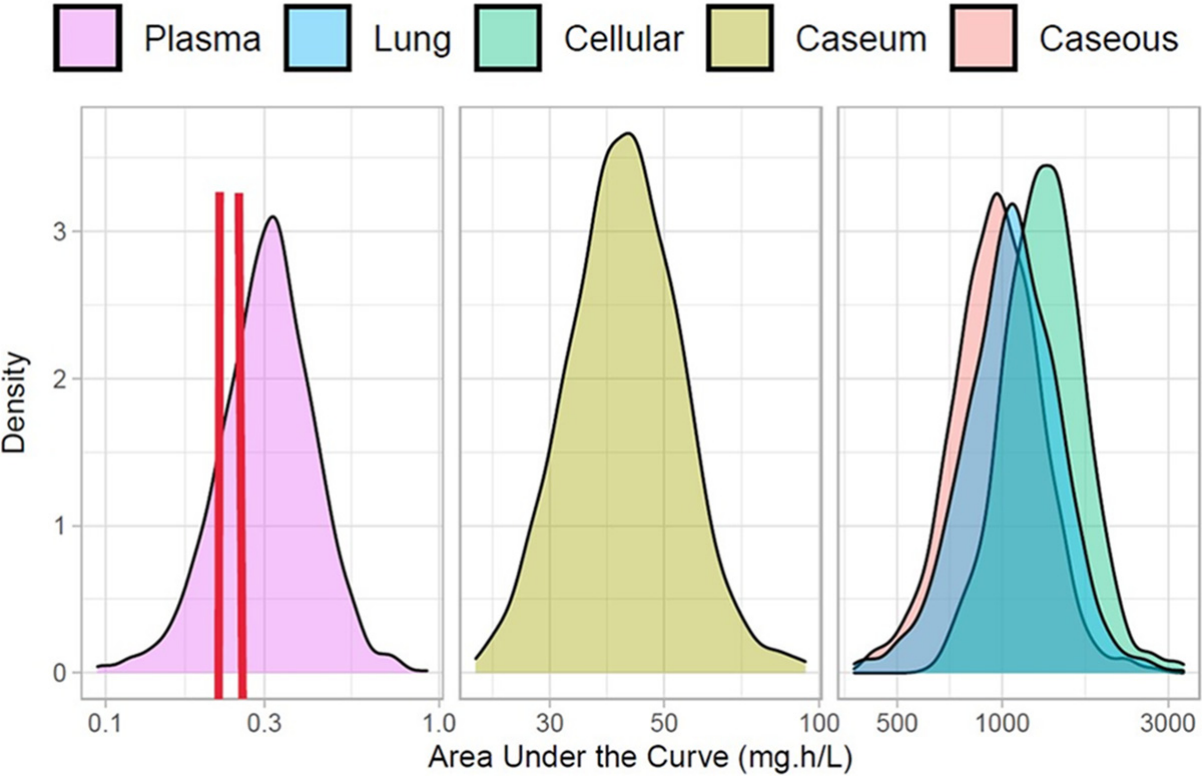
Density plots of steady-state AUC_0-–24_ distributions in plasma, caseum, cellular lesions, caseous lesions, and uninvolved lung tissues from 1,000 simulated rabbits receiving the human-equivalent dose of 25 mg/kg SQ109. The *y* axis is the probability density function (relation between observations and their probability) for the respective AUC in each compartment (*x* axis). The dotted red lines and shaded window show the mean plasma AUC_0–24_ in TB patients after 7 and 14 daily doses of 300 mg in the phase IIa trial (22).

Next, we calculated the median steady-state AUC from 0 to 24 h (AUC_0–24_) and *C*_max_ based on simulations in 1,000 rabbits that received 7 daily doses of 25 mg/kg. The plasma AUC_0–24_ was within the range published for TB patients receiving 300 mg in a phase IIa trial (240.8 ng · h/ml, range 58.4 to 666.5, on day 7 and 268.5 ng · h/ml, range 111.6 to 989.4 on day 14 [22]). The total AUC and *C*_max_ in uninvolved lung tissue, cellular lesions, and caseous lesions were about 3,000-fold higher than the total plasma AUC_0–24_ (Fig. 6 and Table 5). Although the total AUC and *C*_max_ in caseum were dramatically lower than those in other lung and lesion compartments, they were 2 orders of magnitude higher than those in plasma, likely due to higher SQ109 binding to caseum macromolecules than to plasma proteins (caseum *f*_u_ of 0.03 to 0.06% versus plasma *f*_u_ of 80 to 90%, or a difference of 1,000 to 3,000). Under the assumption that free drug concentrations equilibrate between compartments at steady state, our results suggest that 7 daily doses are not sufficient to achieve steady-state concentrations in nonvascularized caseum. Diffusion of the small free fraction into caseum may continue well beyond 7 daily doses, despite steady state being reached in plasma. Multiweek dosing to test this hypothesis was not pursued, as it would lead to resolution of necrotic lesions, preventing representative sampling of caseum. Collectively, our results, model, and simulations indicate that SQ109 achieves PD targets in lung tissue, cellular lesions, and the cellular rims of necrotic lesions and follows a steep declining concentration gradient into necrotic/caseous foci.

**TABLE 5:** SQ109 AUC_0–24_ and *C*_max_ estimates in 1,000 simulated adult female New Zealand white rabbits after 7 daily doses of 25 mg/kg^*a*^

Compartment	AUC_0–24_ (mg · h/liter)		*C*_max_ (mg/liter)	
	Estimate	CV%	Estimate	CV%
Plasma	0.309	32	0.02	18
Cellular	1,319	27	59	26
Caseous	964	30	45	30
Lung	1,073	31	54	33
Caseum	42	26	1.8	25

a CV%, percent coefficient of variation.

## DISCUSSION

SQ109 is a clinical candidate that exhibits several attractive properties; it is active against replicating and nonreplicating M. tuberculosis, is efficacious in animal models and in MDR-TB patients (9–11, 22, 24), and has no demonstrated resistance *in vitro* or *in vivo*. A hallmark of TB is the remarkable ability of the pathogen to persist in necrotic lesions and cavities where both drug penetration and phenotypic drug susceptibility are reduced (31, 32, 48). To guide the design of large phase III trials and prioritize SQ109-containing regimens that achieve optimal lesion coverage, we have profiled its distribution into lesions (PK) and its activity against typical bacterial populations found in cellular and necrotic lesions (PD). Lesion-centric PK-PD analyses predict that SQ109 will mostly contribute activity against bacterial populations present in cellular lesion compartments, where it penetrates at concentrations orders of magnitude higher than required to inhibit growth and kill intracellular and extracellular M. tuberculosis bacilli. In cavity caseum and in the necrotic foci of closed lesions, PK-PD targets were not achieved after seven daily doses. However, SQ109 may diffuse into caseum over several weeks, as observed for the similarly hydrophobic drugs bedaquiline and clofazimine (V. Dartois, F. Kaya, and M. Zimmerman, unpublished). This uncertainty is further compounded by the instability of SQ109 during sample processing for LCM analysis. Additional studies with a panel of hydrophobic TB drugs are required to (i) model the slow creep of these agents and SQ109 in nonvascularized caseum as a function of physicochemical properties and *in vitro* PK profile, (ii) calculate PK-PD indices at steady state, and (iii) determine whether SQ109 can reach steady-state bactericidal concentrations in caseum at tolerated doses.

A two-compartment model with first-order elimination best described SQ109 plasma concentrations, consistent with previous reports (34). Rabbit clearance and volume of distribution were within the range reported for rodents and dogs (33). The model predicted slow oral absorption and oral bioavailability (2.6%) at the lower end of the range reported for mice, rats, and dogs (2.4 to 12% [33]). We next developed a semimechanistic model to characterize the penetration of SQ109 into human-like lesions of TB-infected rabbits. SQ109 concentrations in uninvolved lung and lesions were modeled using effect compartments, as described for other TB drugs (27, 30, 32, 47), showing accumulation in cellular lesions and uninvolved lung tissue in excess of 1,000-fold compared to plasma. This accumulation in regions of high infiltration of immune cells was more pronounced than that measured in noninfected mouse lung (34), consistent with the high partitioning into WBC. *In vitro* uptake in THP-1-derived macrophages, while at the high end of the spectrum observed for other TB drugs (29) and on par with bedaquiline, was ∼50-fold lower than partitioning into WBC. This may be due to (i) differences in small molecule uptake and transport mechanisms between THP-1 and WBC and (ii) differences in protein binding in the two assays, whereby SQ109 protein binding is higher in whole blood than in THP-1 culture medium, leading to enhanced uptake of drug-protein conjugates by phagocytic cells compared to free drug. The latter hypothesis is supported by the general observation that macrophage uptake of small molecule drugs is correlated with protein binding (49).

Comparison of SQ109 concentrations measured in tissue samples collected by LCM versus regular dissection and homogenization revealed a systematic bias toward lower concentrations in LCM samples than in homogenized tissue and lesions. Despite significant efforts to minimize processing time and optimize drug recovery for LCM samples, SQ109 concentrations remained approximately 10-fold lower in the cellular rim of lesions collected by LCM compared to homogenized whole cellular lesions. The reason for the discrepancy could be related to instability during the LCM process at room temperature and/or poor extraction efficiency. Indeed, SQ109 proved to be highly unstable in *ex vivo* caseum, with less than 1% remaining after 7 days. Since LCM is the only approach enabling SQ109 quantitation in caseum, we applied a scaling factor of 10 to the LCM data set for cellular rims and caseum areas. Despite this adjustment, SQ109 concentrations in caseum remained low after 7 doses, the present study duration, compared to PD targets, particularly when corrected for caseum binding. Two limitations thus contribute to uncertainty in estimating SQ109 concentrations in caseum in this study: (i) the 7-day dosing which likely came up short of steady state in tissues and may have contributed to underestimating SQ109 concentrations in caseum at steady state in tissues and (ii) the loss of SQ109 observed during LCM sample processing which led us to introduce a correction factor to compensate for instability and/or poor recovery. Multiweek dosing is not a productive approach in TB lesion PK studies, since drug treatment leads to resolution of pathology, such as necrotic lesions and cavities, preventing representative sampling of the most relevant sites of disease.

The dynamics of hydrophobic drug penetration into caseum is complex. Lipophilic small molecules are typically highly bound to proteins (49) and are highly taken up by macrophages and other immune cells (29, 50), resulting in a low free fraction available for passive diffusion into the avascular caseous core of necrotic lesions and cavities (35). Despite these *in vitro* observations, one cannot exclude slow yet effective equilibration of SQ109’s free plasma concentrations in caseum as expected for highly lipophilic permeable drugs (49), and this may not have been captured after 7 daily doses. The relatively long equilibration half-life (Table 4) into lesions and, particularly, caseum, compared to other TB drugs (32), is consistent with slow creep into caseum. In addition, the high accumulation of SQ109 in immune cells and particularly in foamy macrophages surrounding the necrotic core may act as a reservoir leading to slow drug release, as shown for bedaquiline (51, 52), a drug suspected to reach steady state in tissues after several months of daily dosing (53, 54). SQ109, like bedaquiline, is a cationic amphiphilic drug (high cLog P [calculated octanol:water partition coefficient] of 7.25 and 6.82 and basic pK_a_ of 8.9 and 6.7 for bedaquiline and SQ109, respectively) involved in phospholipidosis, which has been linked to the differentiation of alveolar macrophages into foamy macrophages upon scavenging of lamellar bodies (55). SQ109 may therefore display the same propensity to interact with phospholipids and accumulate in the lipid droplets of foamy macrophages. This would be consistent with SQ109’s equal potency against M. tuberculosis in hypoxic foamy macrophages, where M. tuberculosis exhibits higher drug tolerance, compared to normoxic less foamy macrophages where M. tuberculosis replicates (38).

Pharmacodynamic observations add a layer of complexity to PK-PD target attainment in caseum. Analysis of sputum from the SQ109 phase IIa trial—using metabolic dyes to sort M. tuberculosis into viable replicating, viable nonreplicating, and nonviable bacteria—demonstrated that the addition of SQ109 to rifampicin prevented the increase in Nile-red-positive persister cells seen in the rifampicin-only arm (24). In the present study, we show that SQ109 synergizes with a panel of TB drugs tested under *in vitro* conditions, leading to dormancy (Table 3). Taking these PK-PD considerations into account, whether SQ109 alone or in combination achieves the concentrations required to kill M. tuberculosis persisters in caseum remains to be determined. Weekly sputum sampling over the entire treatment duration would help answer the question of whether SQ109 slowly accumulates in airway fluids, as a surrogate of cavity caseum, and for how long, and would further our understanding of long-term PK-PD target attainment in cavity caseum and sputum. One alternative approach leverages a panel of TB drugs that span a spectrum of physicochemical and *in vitro* PK properties to predict partitioning at the caseum/cellular interface as well as the weekly rate of diffusion into caseum for hydrophobic drugs.

Our results also shed light on the limited efficacy of SQ109 alone in the 14-day EBA trial (22), where the efficacy read-out is the daily decrease of bacterial burden in sputum, which in part drains cavitary material and associated bacteria. Sputum may not be where SQ109’s activity is best revealed, given its slow and limited diffusion into deep caseum. Even in nonnecrotic granuloma mouse models, SQ109 displayed delayed onset of action, requiring 8 to 12 weeks of treatment to observe its maximum effect (21). Like SQ109, bedaquiline and clofazimine are highly bound to caseum macromolecules (35), extensively distributed into vascularized tissue and lesion areas (32, 36), and showed little to no activity in 14-day EBA trials (56, 57). Yet bedaquiline makes critical contributions to novel second-line regimens (58), and clofazimine is included in numerous ongoing treatment-shortening clinical trials (59) based on *in vitro* and preclinical data. Therefore, our results can guide the design of new SQ109-containing regimens based on successful trials that included drugs with similar lesion penetration profiles.

## MATERIALS AND METHODS

### Ethics.

All animal studies were performed in biosafety level 2 (BSL2) and BSL3 facilities and approved by the Institutional Animal Care and Use Committee of the New Jersey Medical School, Rutgers University, Newark, NJ, and Hackensack Meridian Health, NJ. All samples collected from M. tuberculosis-infected animals were handled and processed in the BSL3 facility in compliance with protocols approved by the Institutional Biosafety Committee of the New Jersey Medical School, Rutgers University, Newark, NJ, and Hackensack Meridian Health, NJ.

### Chemical reagents.

SQ109 was provided by Sequella, Inc. (Rockville, MD). SQ109-d4 was synthesized by the group of Clifton E. Barry, NIH-NIAID (Bethesda, MD). Linezolid, moxifloxacin, and bedaquiline were purchased from Sigma-Aldrich (St. Louis, MO), Chem-Impex (Woodale, IL), and Chem Shuttle (Hayward, CA), respectively. Linezolid-d8 and moxifloxacin-d4 were purchased from Toronto Research Chemicals (North York, ON), and bedaquiline-d8 was purchased from Clear Synth (Mumbai, India). All antibiotics tested in pairwise combinations were purchased from Sigma-Aldrich.

### *In vivo* pharmacokinetics in naive and TB-infected rabbits.

Female New Zealand white (NZW) rabbits (Charles River Laboratories, Canada) were used for the pharmacokinetic studies. They were maintained under specific-pathogen-free conditions and fed water and chow *ad libitum*. For intravenous pharmacokinetic studies, rabbits received a single 5- mg/kg bolus dose of SQ109 formulated in 2% ethanol and 98% 0.9% saline in water administered through the marginal ear vein. For dose-finding pharmacokinetic studies in uninfected animals, rabbits received a single oral dose of SQ109 at 22 or 40 mg/kg, formulated in 0.5% carboxymethyl cellulose (CMC) and 0.5% Tween 80 in water and administered by oral gavage. For tissue pharmacokinetics in TB-infected animals, NZW rabbits were infected with M. tuberculosis HN878, using a nose-only aerosol exposure system as previously described (60). At 14 to 20 weeks postinfection, once mature cellular and necrotic lesions had developed, rabbits received one or seven oral daily doses of SQ109 at 25 mg/kg, unless indicated otherwise. Blood was collected from the central ear artery of each rabbit predose and at several time points between drug administration and necropsy (typically, 0.5, 1, 2, 4, 6, and 24 h following drug administration, until the time of euthanasia). Groups of 4 to 5 rabbits were euthanized at 2, 6, and 24 h postdose. All blood samples were centrifuged at 4,000 rpm for 5 min, and the supernatants (plasma) were transferred and stored at −80°C until analyzed by high-pressure liquid chromatography coupled to tandem mass spectrometry (LC/MS-MS).

### Lesion dissection and processing.

From each lung lobe, individual granulomas, mediastinal lymph nodes, and uninvolved (nondiseased) lung tissue areas were dissected, sized, weighed, and recorded. Special care was taken to remove the uninvolved lung tissue surrounding each granuloma. The samples were classified as lymph node, uninvolved lung, or necrotic or cellular granuloma. When necrotic granulomas were greater than 7 mm, they were dissected so that the lesion wall and the caseous material within could be stored and analyzed separately. Lesions collected for laser-capture microdissection and MALDI mass spectrometry imaging were left embedded in the surrounding tissue and snap-frozen in liquid nitrogen vapor as described previously (46). All samples were stored in individual 2-ml tubes at −80°C. Prior to drug quantitation by LC-MS/MS, all tissue samples were homogenized in approximately, but accurately recorded, 4 volumes of phosphate-buffered saline (PBS). Homogenization of tissue samples was achieved using a FastPrep-24 instrument (MP Biomedicals) and 1.4-mm zirconium oxide beads (Precellys). Lung and lesion homogenates were stored at −80°C prior to SQ109 quantitation by LC-MS/MS analysis.

### Binding assay in caseum surrogate.

The caseum binding assay was carried out by rapid equilibrium dialysis using a disposable rapid equilibrium dialysis (RED) device (Thermo Fisher Scientific, MA) as previously described (35). Briefly, caseum surrogate was diluted 10-fold in PBS, homogenized, and spiked at a final incubation concentration of 5 μM. The spiked matrix was placed in the sample chambers, and the buffer chambers were filled with 350 μl of PBS. The plates were then covered with adhesive seals and incubated at 37°C for 4 h on an orbital shaker set at 300 rpm. Following incubation, samples were removed from both chambers and processed by the addition of an organic solvent mixture (1:1 methanol-acetonitrile) prior to LC-MS/MS analysis. The fraction unbound (*f*_u_) in plasma and diluted caseum surrogate was calculated as the ratio between free (buffer chamber) and total (sample chamber) drug concentrations (35).

### Macrophage uptake assay.

Drug penetration assays in human THP-1 cells were performed as previously reported (61). Briefly, THP-1 cells (ATCC TIB-202), grown in RPMI 1640 medium supplemented with 10% fetal bovine serum and 2 mM l-glutamine in a CO_2_ incubator, were seeded into wells of a 96-well tissue culture-treated plate at 5 × 10^4^ cells/well. THP-1 monocytes were differentiated overnight to macrophages with 100 nM phorbol 12-myristate 13-acetate (PMA). Culture medium was carefully removed, and medium containing 5 μM SQ109 was added. Three control drugs were included in the experiment, as representatives of low (linezolid), medium (moxifloxacin), and high (bedaquiline) uptake into macrophages. After 30 min at 37°C, the cells were gently washed twice with ice-cold PBS to remove extracellular drug. Cells were lysed with deionized water for 1 h at 37°C. The drug content of cell lysates was analyzed by LC/MS-MS and subsequently normalized to the number of cells per well (enumerated as described in reference 61) and the average cellular volume to calculate the intracellular concentration of SQ109. The drug accumulation factor is expressed as a ratio between the intracellular concentration and extracellular concentrations (IC/EC) after 30 min of drug incubation.

### Blood cell partitioning.

The blood partitioning assay was conducted using freshly packed whole human blood from the New York Blood Center. Whole blood was spiked with SQ109 at a final concentration of 500 nM (dimethyl sulfoxide [DMSO] content, <1%). After a 1 h of incubation at 37°C and 100 rpm, the whole blood preparation was diluted 1:1 with phosphate-buffered saline (PBS; Gibco, USA). Then, 20 ml of diluted blood was layered over 15 ml of Ficoll (GE Healthcare, Sweden). After centrifugation for 30 min at 530 × *g* and 20°C, 1-ml samples of the top plasma layer, 1-ml samples of the mononuclear cell layer between Ficoll and plasma, and 1-ml samples of the bottom red blood cell (RBC) layer were collected. Mononuclear cell samples were resuspended in PBS and centrifuged at 500 × *g* for 15 min. The cell pellet was further resuspended in PBS and centrifuged again at 100 × *g* for 10 min to remove platelets. The resulting cell pellet contained isolated mononuclear white blood cells (WBC). The exact number of WBCs and RBCs in the samples collected were counted with a hemocytometer. All samples were frozen at −80°C prior to extraction with methanol containing the SQ109-d4 internal standard and LC/MS analysis using the SQ109 quantitation method described below. Plasma SQ109 concentrations were corrected for the 2-fold dilution in PBS. WBC and RBC intracellular concentrations were calculated as follows: SQ109 concentrations were corrected for dilution in the extraction solvent mix and normalized to the number of cells per sample to derive the absolute amount of drug per cell. Intracellular SQ109 concentrations were calculated using reported WBC (1.87 × 10^2^ μm^3^) (62) and RBC (0.87 × 10^2^ μm^3^) (63) cellular volumes. Data are presented as the means of three technical replicates. Intracellular/extracellular concentration (IC/EC) ratios after 30 min of drug incubation were calculated by normalizing to the initial drug incubation concentration.

### Intracellular M. tuberculosis potency assays.

To measure SQ109 activity against intracellular M. tuberculosis, THP-1 monocytes were cultured as described above, differentiated to macrophages with PMA on 24-well cell culture-treated plates seeded with 5 × 10^5^ cells/well. The macrophages were infected with the Erdman strain of M. tuberculosis at a multiplicity of infection (MOI) of 1:1. After 4 h of infection, the wells were washed three times with PBS to remove extracellular bacteria. Fresh medium containing 0.5, 1, 2, 4, or 8 μM SQ109 was added, with vehicle-only wells included as controls. After 3 days at 37°C and 5% CO_2_, the THP-1 macrophages were detached with 5 mM EDTA and lysed with 0.05% sodium dodecyl sulfate (SDS), and serial dilutions of the lysates were plated on Middlebrook 7H11 agar for CFU enumeration.

To measure SQ109 activity against intracellular M. tuberculosis in primary macrophages, human peripheral blood monocytes (PBMCs) were isolated from freshly packed human blood (New York Blood Center) by Ficoll separation followed by purification using the EasySep human CD14 positive selection kit II according to the manufacturer’s instructions (Stemcell Technologies, Canada). Approximately 5 × 10^4^ cells in 200 μl medium were plated and incubated at 37°C and 5% CO_2_ for 3 days in RPMI supplemented with 10% human serum, 200 ng/ml macrophage-colony stimulating factor (M-CSF), 100 IU/ml penicillin, and 100 mg/ml streptomycin. To complete differentiation of monocytes into macrophages, the cell culture medium was replaced with fresh, antibiotic-free medium containing M-CSF, and cells were cultured under identical conditions for another 4 days. Macrophages were infected with M. tuberculosis Erdman constitutively expressing the mCherry far-red fluorescent protein (64) at a multiplicity of infection of 1:1, under normoxic and hypoxic conditions as described in reference 38. On day 3 postinfection, SQ109 was added from 0.001 to 20 μM, and infected macrophages were incubated for another 5 days with medium and drug replacement on day 3. mCherry fluorescence signals were measured at excitation/emission wavelengths of 572/610 nm, and all readings were corrected for background fluorescence. EC_50_ and EC_90_ were defined as the effective concentrations that reduced mCherry fluorescence signal by 50% and 90%, respectively, compared to the day 5 untreated control.

### Pharmacodynamic interaction measurements.

Pairwise drug interactions with SQ109 were measured using diagonal measurement of *n*-way drug interaction (DiaMOND) experimental design and analysis (43). Measurements were made using M. tuberculosis Erdman carrying an auto-luminescent reporter (pMV306hsp+LuxG13; Addgene) and were maintained using 25 μg/ml kanamycin. M. tuberculosis was adapted to medium before seeding for the DiaMOND assay into 384-well plate format as follows—standard (rich): 7H9 supplemented with 0.2% glycerol, 10% oleic acid-albumin-dextrose-catalase (OADC), and 0.05% Tween 80; butyrate, cholesterol, cholesterol (high), and valerate: 7H9 with fatty acid-free bovine serum albumin (BSA) (0.5 g/liter), NaCl (100 mM), tyloxapol (0.05%), and MOPS (morpholinepropanesulfonic acid; 100 mM) to adjust to pH 7 and then supplemented with sodium butyrate (0.1% wt/vol, final concentration), valeric acid (0.1% vol/vol, final concentration), or cholesterol (0.05 mM or 0.2 mM, final concentration after dissolving cholesterol in a 1:1 ethanol and tyloxapol mixture; dormancy: butyrate medium supplemented with sodium nitrate (5 mM) and sealed in multiwell plates to limit oxygen and induce dormancy. Antibiotics were dissolved in DMSO and dispensed using the HP 300e digital drug dispenser. For the dormancy model, luminescence was measured after 6 days in recovery (standard) medium following 7 days of treatment in dormancy conditions. The optical density at 600 nm (OD_600_) (all other models) was measured at 5 (standard), 10 (butyrate), 15 (valerate), 28 (cholesterol), and 24 (cholesterol-high) days posttreatment. Dose responses were measured in 10-dose resolution. To calculate dose responses, background value (median OD/luminescence of medium-only containing wells) was subtracted from raw OD/luminescence reads. Dose responses of drug-treated wells were normalized to untreated wells and were fitted to a three-parameter Hill equation to extract IC values along the curve. Fractional inhibitory concentrations (FICs) at the indicated inhibition level (FIC_90_ is at IC_90_ and FIC_75_ is at IC_75_) were determined using Loewe additivity as the null model as previously described (43). We reported FIC_75_ in conditions where many antibiotics did not reach IC_90_.

### LC-MS/MS method for quantitation of SQ109 in plasma and tissue homogenates.

Neat 1 mg/ml DMSO stocks for SQ109 were serial diluted in 50/50 acetonitrile (ACN)/Milli-Q water and subsequently diluted in drug free control tissue homogenates or plasma for creation of the standards and quality control (QC) samples. SQ109 standard and SQ109-d4 internal standard (IS) were received from Sequella. New Zealand white (NZW) rabbit control plasma (K_2_EDTA; BioIVT, New York) and γ-irradiated lung, lesion, and caseum from TB-infected NZW rabbits were used to build standard curves. Control and study sample lung tissues were homogenized by adding 9 parts PBS buffer as follows: 1 part tissue (10× dilution) and shaking the samples using a Fisher bead mill for 1 min at 6,000 rpm with zirconia beads. Standards, QCs, controls, and study samples were extracted by combining 20 μl of study sample, QC, standard, or control with 200 μl 50/50 ACN/methanol (MeOH) containing 20 ng/ml of the IS. Extracts were vortexed for 5 min and centrifuged at 4,000 rpm for 5 min. Then, 100 μl of supernatant was transferred to a 96-well plate for LC-MS/MS analysis and was diluted with 100 μl of Milli-Q water.

LC-MS/MS analysis was performed on a Sciex Applied Biosystems QTRAP 6500+ triple-quadrupole mass spectrometer coupled to a Shimadzu Nexera X2 ultra-high-performance liquid chromatography (UHPLC) system to quantify each drug in plasma. Chromatography was performed on an Agilent Zorbax SB-C8 column (2.1 × 30 mm; particle size, 3.5 μm) using a reverse-phase gradient elution. Milli-Q deionized water with 0.1% formic acid was used for the aqueous mobile phase, and 0.1% formic acid in ACN was used for the organic mobile phase. Multiple-reaction monitoring (MRM) of precursor/fragment transitions in electrospray positive-ionization mode was used to quantify the analytes. MRM transitions of 331.2/178.2 and 335.2/182.2 were used for SQ109 and IS, respectively. Sample analysis was accepted if the concentrations of the quality control samples were within 20% of the nominal concentration. The lower limit of quantification (LOQ) was 1 ng/ml in plasma and 10 ng/ml in lung tissues. Sample analysis was accepted if the concentrations of the quality control samples were within 20% of the nominal concentration, and the coefficient of variation (%CV) of the QC replicates was less than 20%. Data processing was performed using Analyst software version 1.6.2 (Applied Biosystems Sciex).

### Laser-capture microdissection.

Laser-capture microdissection (LCM) was carried out as previously described (46). Briefly, γ-irradiated frozen lung biopsy specimens were sectioned at 10 μm for histology and 25 μm for LCM using a CM1810 cryostat (Leica). Sections for histological analysis were taken immediately adjacent to those taken for LCM, and data were correlated. LCM sections were thaw-mounted onto 1.4-μm thick polyethylene terephthalate (PET) membrane slides (Leica). Regions of necrotic caseum, their corresponding cellular rim, and normal lung tissue, were dissected using an LMD7 microscope (Leica) until an area of 3 million μm^2^ was collected for each region. Dissected regions of interest were stored at −80°C until analysis.

SQ109 concentrations measured in thin-section samples collected by LCM were systematically lower than concentrations measured in lesion homogenates of the same rabbits. Despite significant efforts to minimize processing time and optimize drug recovery for LCM samples, SQ109 concentrations remained on average 10-fold lower in the cellular rim of lesions collected by LCM compared to homogenized whole cellular lesions, and approximately 2.5-fold lower in uninvolved lung collected by LCM compared to homogenized uninvolved lung. The pharmacokinetic model was used to generate estimates of these two scaling factors. The reason for the discrepancy could be related to instability (which has been observed in caseum) during the LCM process at room temperature and/or lower extraction efficiency from LCM samples than tissue homogenates. We applied a scaling factor of 10 to the LCM data set for cellular rims and caseum areas and a scaling factor of 2.5 for uninvolved lung tissue. We indicate whether these scaling factors are applied to the data shown in the Results, graphs, and figures.

### LC-MS/MS method for quantitation of SQ109 in laser capture microdissected samples.

LCM sample quantification was carried out according to a previously published protocol (46). Briefly, 1 mg/ml DMSO stocks of SQ109 were serial-diluted in 50/50 ACN/Milli-Q water to create neat standards. Control tissue homogenate was created by adding 25.6 parts PBS buffer as follows: 1 part tissue (26.7× dilution) and shaking the samples using a Fisher bead mill for 1 min at 6,000 rpm with zirconia beads. Standards, quality control, and control samples were extracted by adding 2 μl of blank homogenate, 10 μl of standard, and 50 μl of extract solvent containing 50/50 ACN/MeOH and IS. Laser-microdissected study samples and standards were extracted using 2 μl of PBS. Extracts were bath-sonicated for 10 min and centrifuged at 4,000 rpm for 5 min. Then, 50 μl of supernatant was transferred to a 96-well plate for LC-MS/MS analysis. LC-MS/MS analysis was performed as described for lesion homogenate analysis.

### MALDI mass spectrometry imaging.

Mass spectrometry imaging experiments were carried out on serial sections taken before and after those collected for LCM. Sections were taken at 10 μm using a CM1810 cryostat (Leica), thaw-mounted onto Superfrost Plus glass slides (Thermo Fisher Scientific), and stored at −80°C until analysis. Upon analysis, slides were removed from the −80°C freezer and desiccated for 15 min prior to matrix deposition. 2,5-dihydroxybenzoic acid (DHB) (25 mg/ml in 50% methanol containing 0.15% trifluoroacetic acid [TFA]) was deposited over the tissue sections using the HTX sprayer (HTX). Spray parameters were as follows: 60°C, flow rate of 50 μl/min, velocity of 1,200, 8 lb/in^2^, and 30 cycles. Imaging acquisition was carried out using a MALDI LTQ XL Orbitrap mass spectrometer (Thermo Fisher Scientific) operated in the positive ion mode with a mass range of *m/z* 185 to 400 and a pixel resolution of 75 μm. All data were processed using ImageQuest software (Thermo Fisher Scientific). Following data acquisition, the matrix was washed from the slides using 70% ethanol, and the sections were stained with hematoxylin and eosin (H&E) according the manufacturer’s protocol (Thermo Fisher Scientific).

### PK data analysis and modeling.

In total, 132 plasma samples and 402 lesion or lung tissue samples were collected and analyzed as follows: 95 uninvolved lung pieces, 50 cellular lesions, and 112 caseous lesions analyzed by mass spectrometry in lesion homogenates and 50 uninvolved lung areas, 47 cellular rims, and 48 caseous foci dissected by LCM. Of these data, 3 plasma and 30 tissues samples were identified as outliers and were removed from the model-building process. Two plasma and thirteen tissue samples had SQ109 concentrations below the lower limit of quantification (LLOQ). The population PK of SQ109 in plasma and lesions was described by nonlinear mixed-effect modeling using the software NONMEM version 7.4.4 (Icon Development Solutions, Ellicott City, MD) and the algorithm first-order conditional estimation with interaction (FOCE-I). Perl-Speaks-NONMEM version 4.7.4, the R software version 3.2.5 and its package xpose4, and Pirana version 2.9.8 (65) were used for graphical processing of NONMEM output and management of the model development process.

The modeling process was carried out in a stepwise manner. First, we developed the structural model to describe plasma concentrations of both orally and intravenously administered SQ109. For this, we evaluated one-, two-, and three-compartment models with first-order elimination. For oral administration, we used first-order absorption with and without a time lag. Allometric scaling was used to adjust for the effect of body size on disposition parameters with allometric exponents fixed to 0.75 for clearance parameters and 1 for volumes of distribution (66). Log-normally distributed random effects were included for the disposition parameters to capture between-subject variability (BSV) and for absorption parameters to describe between-occasion variability (BOV). The oral bioavailability parameter was estimated to describe the PK of orally administered SQ109.

Second, we included in the model tissue concentrations determined in tissue homogenates (uninvolved lung tissues and cellular and caseous lesions). The concentration in tissue was described using an effect compartment approach (27, 47). This approach entails that, while the drug concentration in the tissue depends on the concentration profile in plasma, the model assumes these sites are negligibly small compared to the total volume of distribution of the drug, so they will not affect the drug concentration in the central compartment. In other words, there is negligible transfer of mass into these effect compartments compared with the amount in plasma and vice versa, but the concentration in the effect compartments is only a reflection of the concentrations in plasma. This is achieved by estimating a time for *C*_plasma_-*C*_tissue_ equilibration rate constant (*K*_tissue_) and a pseudopartition coefficient (*R*_tissue_), as shown in equation 1:

(1) $$\frac{dC_{\mathrm{tissue}}}{\mathrm{dt}}=K_{\mathrm{tissue}}\times \left(R_{\mathrm{tissue}}\times C_{\mathrm{plasma}}-C_{\mathrm{tissue}}\right)$$

Equation 1 focuses on obtaining the amount of SQ109 in each of the tissues (cellular and caseous lesions, uninvolved lung tissue, and caseum) such that *K*_tissue_ represents a first-order rate constant of the transfer of drug from plasma to tissue or lesion. The equilibration half-life *In*(2)/*K*_tissue_ describes the time required to achieve 50% of the effect compartment equilibrium target, assuming plasma levels are at steady state. *R*_tissue_ is the pseudopartition coefficient into the respective tissue compartment. *C*_plasma_ is the concentration in the central compartment. Equation 1 applies to a theoretical compartment with no volume and no transfer of drug from this compartment back to central compartment.

Third, since we have SQ109 concentration measurements generated with LCM samples and lesion homogenates for cellular lesions and uninvolved lung, we used the same effect compartments to describe both measurements and introduced scaling factors to account for the difference between the LCM and the traditional LC-MS/MS applied to homogenates. For caseum samples, only LCM data were available, and we applied the scaling factor estimated from cellular samples to adjust for the difference between the two methods. In the final model, we estimated all the parameters for both plasma and lesions simultaneously. The residual unexplained variability for each observation was described using a combined error model with a proportional and an additive component, the latter which was constrained to be at least 20% of the assay lower limit of quantification. A minor modification to the original implementation of the M6 method (67) was used to handle concentration below the LLOQ. For the censored concentrations that have been substituted with LLOQ/2, the additive error is increased, thus mitigating the impact of the value used for the imputation. By increasing the size of the additive error, we make sure the imputed value for that observation carries less weight while still preserving the information that a low concentration was observed at that time point. Based on graphical exploration confirmed by the absolute value of conditional weighted residuals (CWRES) greater than 4, implausible concentrations were identified and removed from the model development process. CWRES are expected to follow a normal distribution with mean 0 and variance 1 for a correct model; hence, less than 0.01% of data is expected to have |CWRES| > 4 to achieve good model fit (68).

The model selection was based on the objective function value (OFV), the plausibility of the parameter values, and the visual inspection of diagnostic plots. The robustness of the final parameter estimates was carried out using the sampling importance resampling (SIR) (69) method with 1,000 samples.

### Pharmacokinetic simulations.

The final model was used to simulate predicted plasma and lesion concentration in each tissue for 1,000 rabbits with median weight of 3.5 kg receiving 25 or 40 mg/kg of SQ109 daily for 7 days to reach the steady state. The predicted concentrations in plasma, uninvolved lung tissues, cellular lesions, and caseous lesions on day 7 were compared to the concentration required to inhibit the growth of or kill M. tuberculosis bacilli in relevant potency assays that represent the major bacterial populations found in lesions. Monte Carlo simulations of plasma and lesion concentration data in 1,000 rabbits for an oral SQ109 dose of 25 mg/kg administered once daily for 7 days were performed to visualize the distribution of AUC and *C*_max_ in density plots where the *y* axis is the probability density function or kernel density estimate for plasma and tissue compartments.
