# Supplementary material for: Pharmacokinetics and Target Attainment of SQ109 in Plasma and Human-Like Tuberculosis Lesions in Rabbits
Source: Antimicrob Agents Chemother. 2021 Aug 17;65(9):e00024-21. doi: 10.1128/AAC.00024-21 (PMC8370215; doi:10.1128/AAC.00024-21)
Supplement: Supplemental file 1 — Supplemental material. Download AAC.00024-21-s0001.pdf, PDF file, 1.1 MB [file aac.00024-21-s0001.pdf]

SUPPLEMENTARY FIGURES

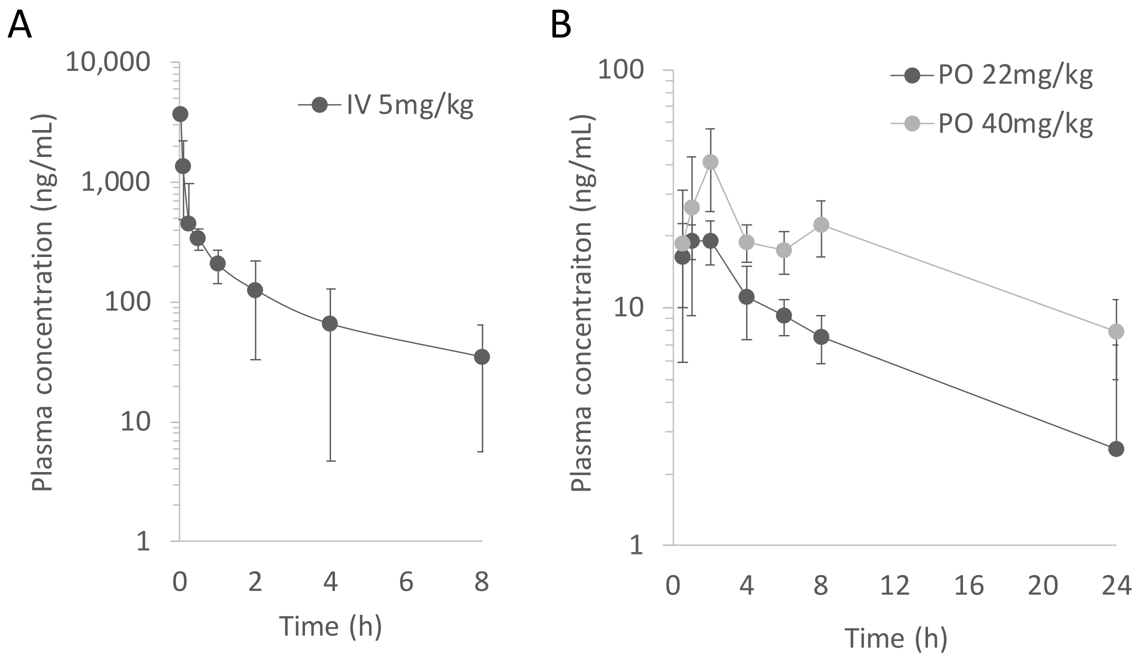

Figure S1. Plasma concentration-time profile of SQ109 in uninfected New Zealand White rabbits, following a single intravenous bolus dose of 5 mg/kg (A) and single oral doses of 22 and 40 mg/kg (B). Mean and standard deviations are shown (n=3).

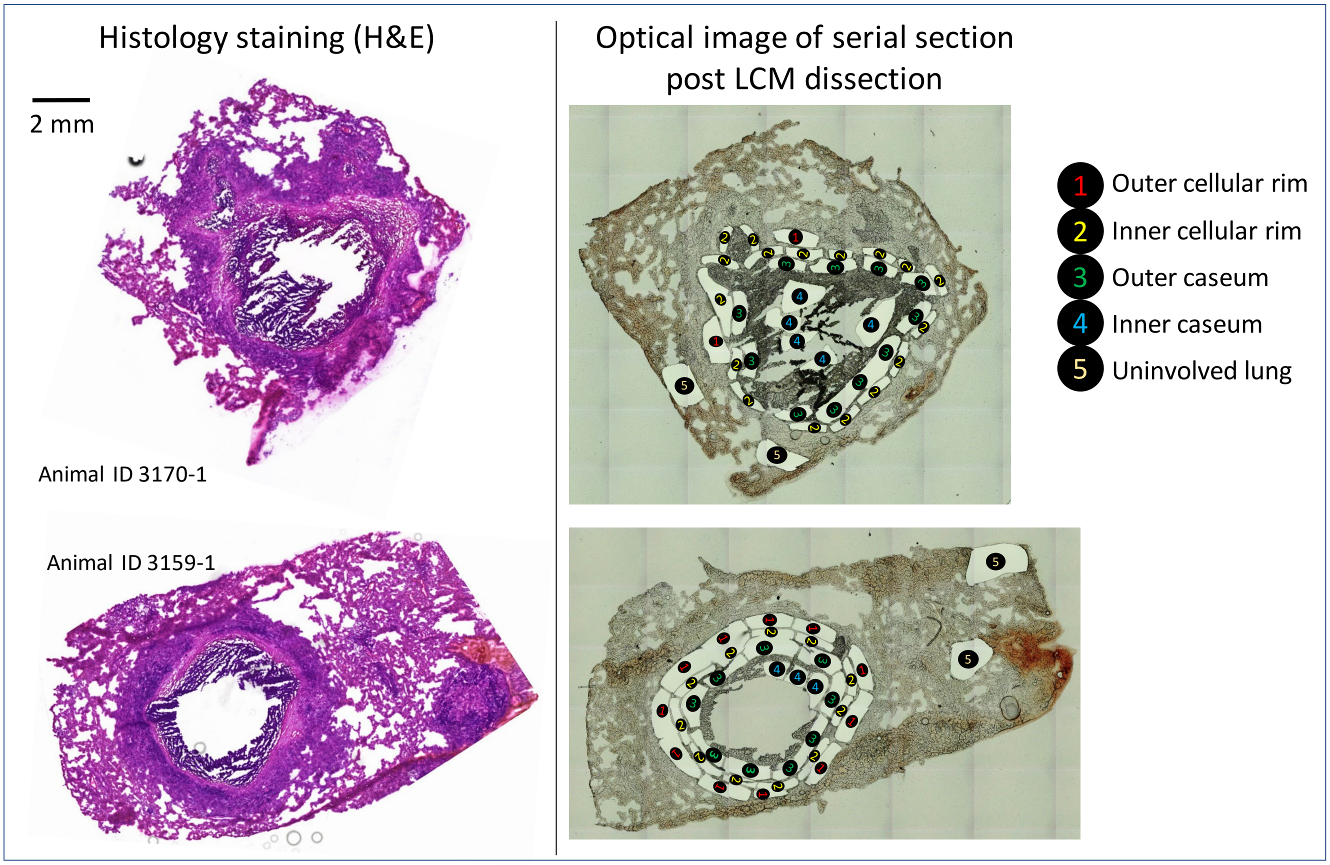

Figure S2. Depiction of the histology staining and laser-capture microdissection (LCM) process. Adjacent lesion sections were used for hematoxylin and eosin (H&E) staining (left) to guide LCM sample collection (right). Two large necrotic lesions were collected from two rabbits 6h after the 7th daily dose of 25 mg/kg. LCM pieces belonging to the same tissue compartment category were pooled for quantitation by LC-MS/MS. The caseous center of large necrotic lesions is friable and was partially lost upon sectioning.

A

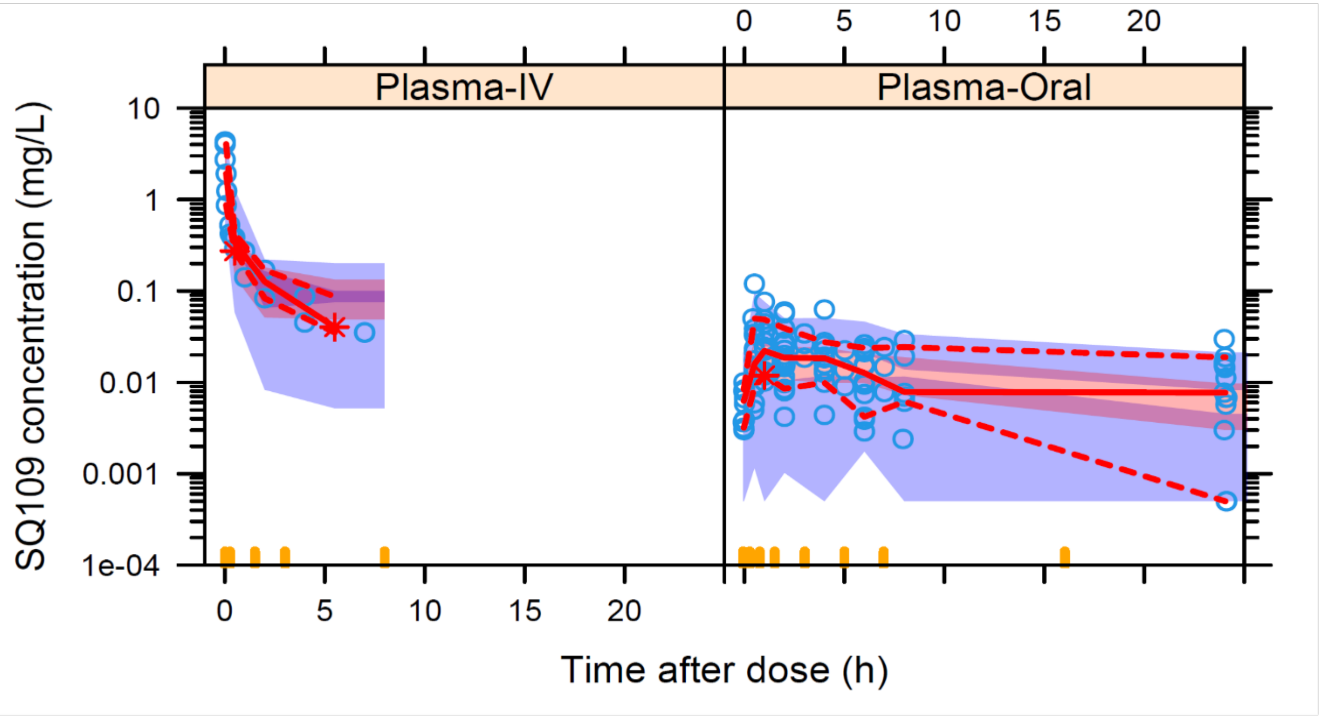

B

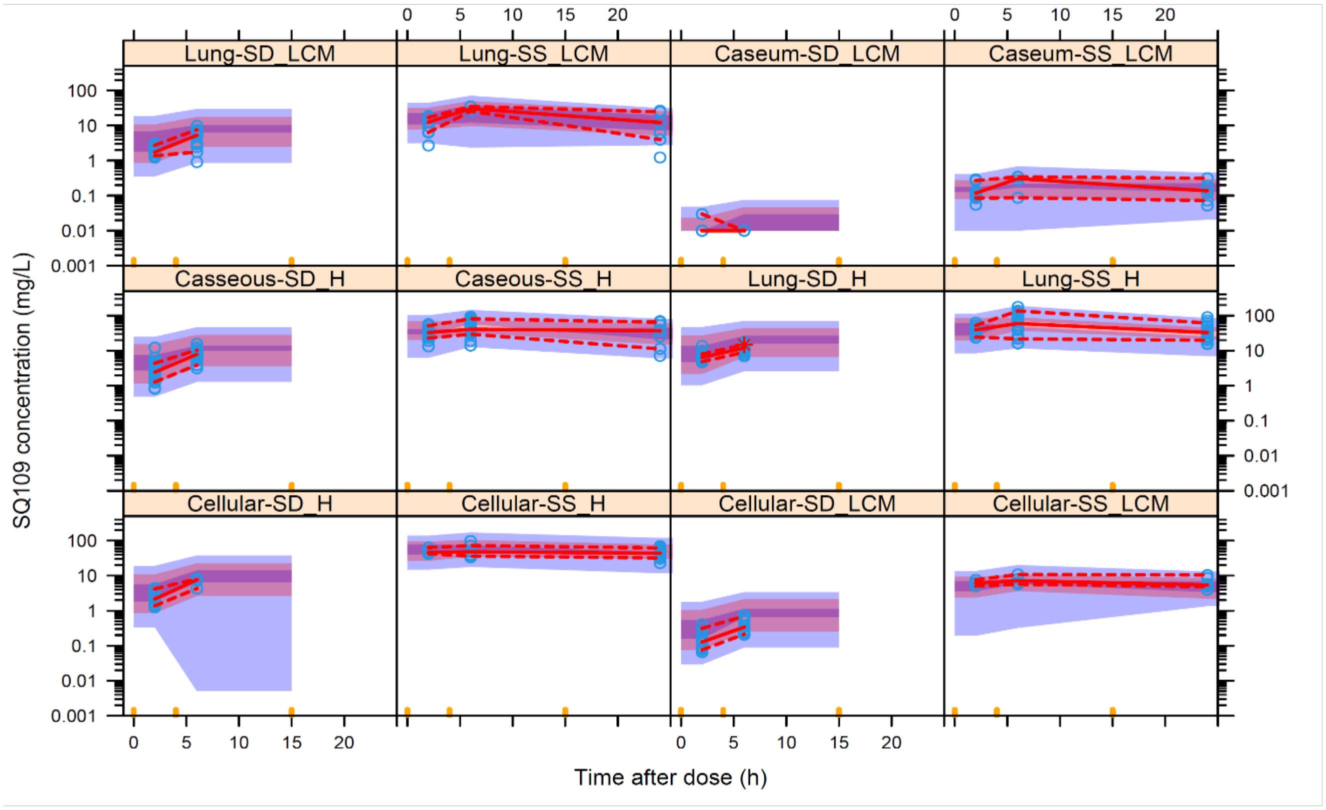

Figure S3. Visual predictive check (VPC) for the final SQ109 population PK model. **(A)** Plasma concentration-time profiles following intravenous and oral administration. **(B)** Concentration-time profile simulations in 1,000 rabbits in lung, cellular and caseous lesions, and caseum, following a single dose (SD) and at steady state (SS).

Modeling of data collected by quantitation of SQ109 in tissue homogenates (H) and in LCM are plotted separately. The scaling factors were estimated and applied by the model. Open circles are observed concentrations. The middle continuous line is the 50th percentile of the observed data, the lower and upper dashed lines are the 5th and 95th percentiles of the observed data, respectively. The shaded regions represent the 95% prediction intervals of the 10th, 50th, and 90th percentiles.
